# Does a Spontaneous Intracerebral Hemorrhage Predispose to a Secondary, Distant Intracerebral Hemorrhage? A Case Report and Review of the Literature

**DOI:** 10.7759/cureus.1999

**Published:** 2017-12-29

**Authors:** Nakul Katyal, Christopher R Newey

**Affiliations:** 1 Neurology, University of Missouri Columbia; 2 Neurology, Cleveland Clinic Ohio

**Keywords:** intracerebral hemorrhage (ich), dysregulation, cerebrovascular system

## Abstract

Cerebrovascular autoregulation may be dysfunctional after acute intracerebral hemorrhage (ICH). This disruption in autoregulation can potentially result in secondary neurological damage that may present as an intracranial hemorrhage at locations distant from the primary site of hemorrhage. We discuss a case of 68-year-old female who presented with acute left hemiparesis from a spontaneous right frontal ICH. Magnetic resonance imaging (MRI) was negative for any other blooming artifact. Her weakness was improving, but after 72 hours from admission, she had an acute change in her mental status and was found to have a new left frontal ICH distant from the primary hemorrhage. Cerebral dysregulation following spontaneous ICH may predispose patients with risk factors, such as chronic hypertension, to a secondary spontaneous ICH distant from initial ICH. Recognizing this phenomenon can guide the management of acute ICH.

## Introduction

Spontaneous intracerebral hemorrhage (ICH) is the second leading cause of stroke and is associated with significant morbidity and mortality [1-2]. Elevated blood pressure is a frequent finding during the acute phase of a spontaneous ICH and is associated with poor outcomes [3-4]. Studies have reported about the development of ischemic lesions, visualized on diffusion-weighted images (DWI), occurring at sites distant to the primary ICH [5]. This finding may be secondary to dysfunctional autoregulation. We present a patient who developed a left frontal ICH several days following a right frontal ICH. We believe the occurrence of the second ICH in our patient was related to dysregulation of cerebral vasculature following the primary ICH. We describe the case and review the literature on cerebrovascular dysregulation following ICH.

## Case presentation

**Figure 1:**
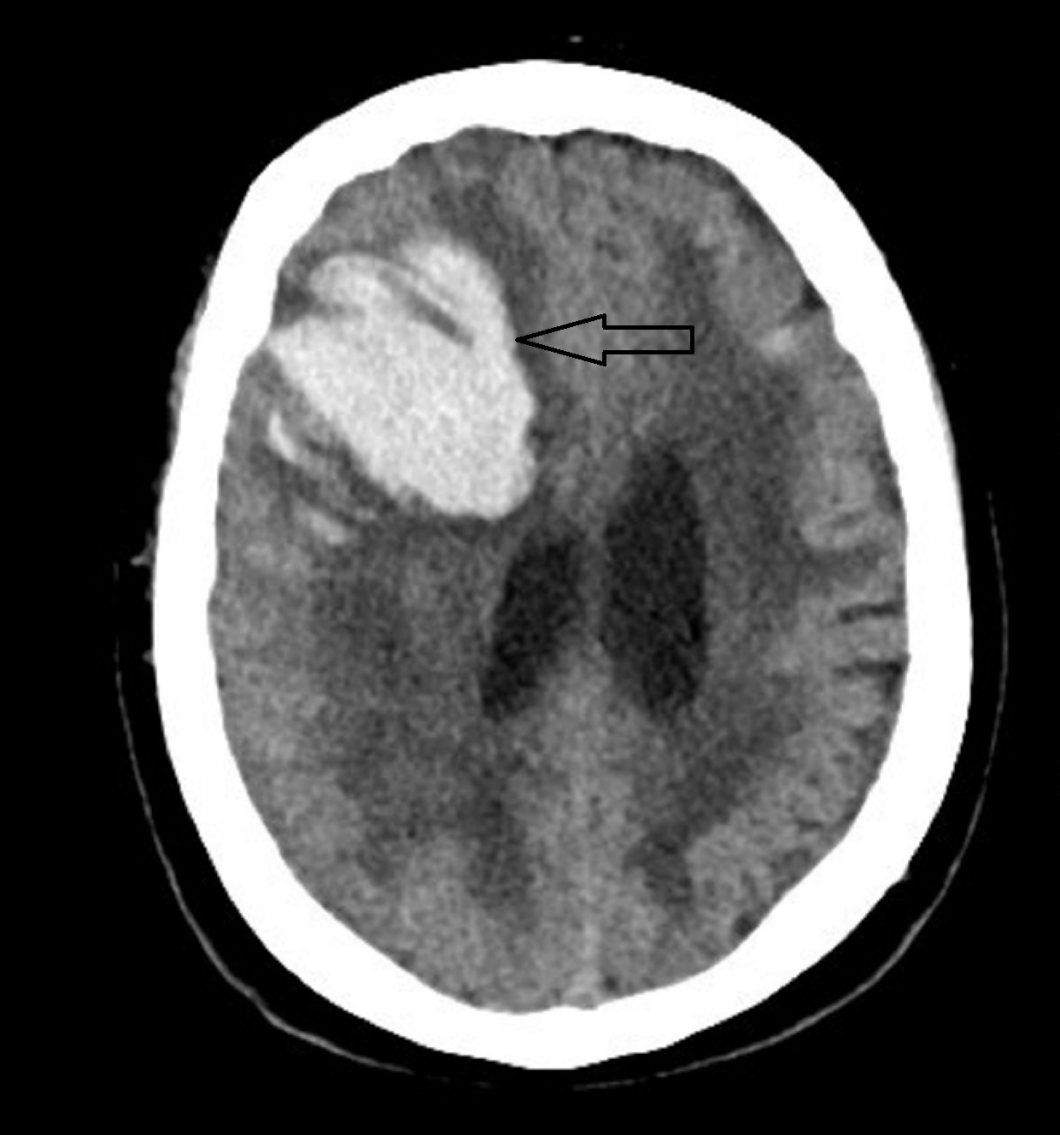
Initial Computed Tomography (CT) Head From Outside Hospital CT shows a right frontal intracerebral hemorrhage (56.5 cc) with mass effect on the right lateral ventricle.

A 68-year-old female with a past medical history of mild cognitive impairment and hypertension was brought to the emergency department (ED) from an outside hospital for evaluation of a right frontal ICH. The patient had left arm and left leg weakness for approximately one day prior to admission. On arrival to the ED, she was somnolent and difficult to arouse. She was hypertensive with a blood pressure of 173/86 mmHg. Her Glasgow Coma Scale (GCS) score was 11 (E:2, V:4, M:5), and her ICH score was 3. On neurological examination, her pupils were equal and reactive to light bilaterally with right-sided gaze preference along with left face, arm, and leg weakness. Computed tomography (CT) head scan from the outside hospital showed a right frontal ICH (4.3 cm x 5.5 cm x 4.7 cm) (Figure 1).

**Figure 2:**
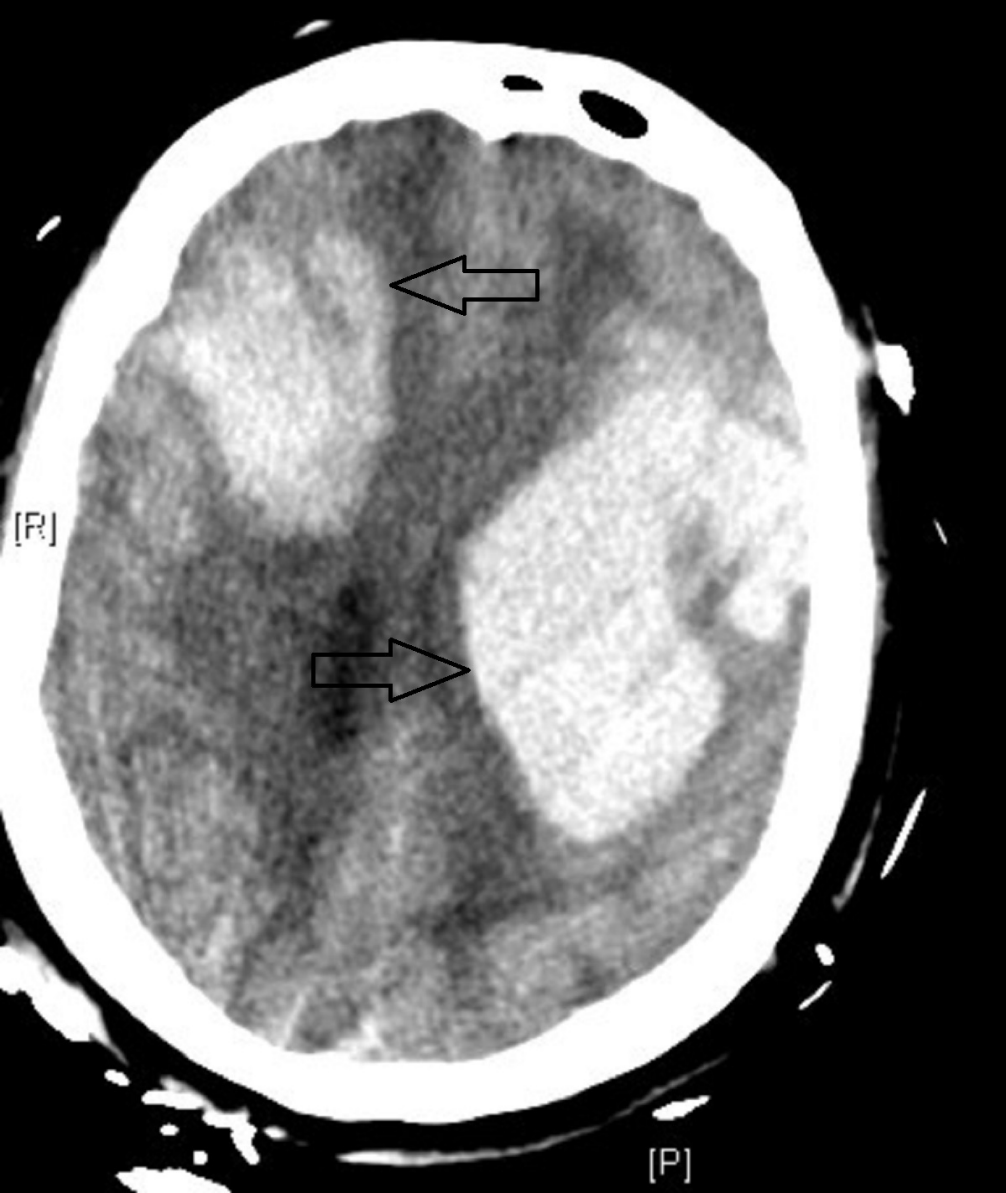
Computed Tomography (CT) Head on Day 9 CT shows the known right frontal intracerebral hemorrhage, but now with new left frontal intracerebral hemorrhage  (47.8 cc).

Computed tomography angiography (CTA) was negative for any underlying vascular etiology for the ICH. International normalized ratio (INR) was 1.1, and platelet count was 209 x 109/L. Her blood pressure was controlled with intravenous nicardipine for a goal systolic blood pressure of < 160 mmHg. She was admitted to the neuroscience intensive care unit (NICU) for further evaluation. A brain magnetic resonance imaging (MRI) scan confirmed a right frontal ICH (4.3 cm x 5.5 cm x 4.7 cm). There were no other areas of blooming artifact on the MRI. There was evidence of small vessel disease in bilateral centrum semiovale. Serial CT head scans showed a stability of the ICH. During her admission to the NICU, she was improving neurologically and was scheduled for acute rehabilitation. However, on Day 9 of admission to NICU, she sustained a generalized tonic-clonic seizure. She was unable to protect her airway and required intubation. CT of the head showed the known right frontal ICH with thin, bilateral cortical sulci subarachnoid and intraventricular hemorrhages. However, now there was a new, large left frontal ICH (5 cm x 3.9 cm x 4.9 cm) (Figure 2).

Her coagulation panel had remained normal throughout her hospitalization. Electroencephalogram (EEG) was negative for seizures or any epileptiform abnormalities. Her neurological exam was persistently poor following the second ICH. Ultimately, the family decided to proceed with comfort care measures. Palliative care was consulted and comfort measures were initiated.

## Discussion

Our case highlights the phenomenon of a new, spontaneous ICH remote from the initial cerebrovascular event. Studies have reported the association of acute blood pressure dysregulations and the development of new ischemic lesions distant from primary ICH [5]. In a multicenter observational study of ICH among 600 patients of different racial backgrounds, Kidwell, et al. reported that new ischemic lesions occurred in 26.5% of the patients following a primary ICH. Lower age, higher first recorded systolic blood pressure, a greater change in mean arterial pressure (MAP) prior to the MRI, microbleeds, and a higher white matter hyperintensity (WMH) score were associated with an increased likelihood for the development of ischemic lesions. Additionally, new ischemic lesions were associated with poorer outcomes [6]. Our patient had increased white matter hyperintensity (WMH) changes in the bilateral centrum semiovales, which likely predisposed her to the second ICH. The pathophysiology of distant cerebrovascular changes have recently been studied. Garg, et al. analyzed brain MRI scans of 95 patients with ICH for evaluation of areas of restricted diffusion. Thirty-eight percent of patients with ICH were found to have restricted diffusion on MRI scan, which had been performed at a median time interval ranging from 1.6 to 2.3 days after symptom onset. Restricted diffusion was found to be associated with a higher incidence of death or dependence at three months. This study hypothesized that acute blood pressure reduction was the cause for the ischemic lesions [7]. In another study, Nakagawa, et al. compared 21 patients with early lobar or basal ganglia ICH with 23 age-matched controls and evaluated the function of cerebral autoregulation. Dynamic cerebral autoregulation was assessed using MAP and mean flow velocity (MFV). This study concluded that cerebral autoregulation may be ineffective in the early days after ICH. Additionally, the mass effect of a hematoma may have a direct effect on dynamic cerebral autoregulation. Larger hematomas have been associated with increased intracranial pressure and lower intracranial compliance. This decreased compliance may impair fast-responding myogenic responses that influence the rapidity of vascular adjustment. Also, higher intracranial pressure may reduce cerebral perfusion pressure, which can significantly impair autoregulation [8]. 

Our patient sustained a spontaneous left frontal ICH after an initial right frontal ICH. We believe she suffered a dual insult of compromised static and dynamic components of cerebral autoregulation that led to the second ICH. She had a longstanding history of hypertension, mild cognitive impairment, and significant small vessel ischemic changes on neuroimaging. Chronic hypertension is well-known to affect the static component of cerebral autoregulation. The cerebral autoregulation curve is shifted to the right in patients with untreated chronic hypertension [8]. The dynamic component of cerebral autoregulation could have been rendered ineffective secondary to the mass effect of an initial right frontal ICH. The mass effect raised the intracranial pressure and decreased the intracranial compliance. A higher intracranial pressure may reduce the cerebral perfusion pressure, which can impair the dynamic component of autoregulation. High blood pressure can also impair the dynamic component, which was seen in our patient at initial presentation. All these factors can well explain the occurrence of a new distant left frontal ICH after an initial right frontal ICH. 

Blood pressure regulation following an ICH has been the focus of several large trials. The Intensive Blood Pressure Reduction in Acute Cerebral Hemorrhage 2 (INTERACT2) trial evaluated 2,839 patients with spontaneous ICHs and elevated blood pressure. This study compared the effectiveness of intensive blood pressure reduction to a systolic blood pressure (SBP) of 140 mmHg versus a guideline-recommended blood pressure reduction to an SBP of 180 mmHg using antihypertensive medications. No significant differences were seen in mortality or disability rates among two treatment groups. However, improved functional outcomes were reported with an intensive lowering of blood pressure [9]. The Antihypertensive Treatment of Acute Cerebral Hemorrhage II (ATACH II) trial was conducted to determine the efficacy of early, intensive antihypertensive treatment using intravenous nicardipine initiated within three hours of onset of ICH and continued for next 24 hours in patients with spontaneous ICH. The primary hypothesis of the expected beneficial effect of intensive treatment was presumably mediated through reduction of the rate and magnitude of hematoma expansion. However, no significant difference was observed in the death or disability rates with intensive blood pressure management as compared to a standard reduction [10]. This highlights how hypertension can affect autoregulation, and the aggressive management of hypertension can predispose to injury.

We presume that small vessel ischemic changes of chronic hypertension predisposed our patient to an increased risk of cerebrovascular dysregulation, which was potentiated after the primary ICH. The finding of ICH at a distant location following a primary ICH can be related to the disruption of cerebrovascular regulatory mechanisms resulting from the double insult of chronic hypertension and ICH.

## Conclusions

Our case highlights the phenomenon of a second ICH from cerebrovascular dysregulation following a primary spontaneous ICH. Recognizing this phenomenon and appreciating potential risk factors may guide the management of patients with acute ICH.
